# Maggot Infestation of the Prolapsed Uterus

**DOI:** 10.7759/cureus.3554

**Published:** 2018-11-06

**Authors:** Muhammad Hamza, Tahira Yasmeen, Noor Fatima, Irfan Ahmed Nadeem

**Affiliations:** 1 Surgery, Al Noor Surgery Hospital, Chakwal, PAK; 2 Gynecology and Obstetrics, Holy Family Hospital, Rawalpindi, PAK

**Keywords:** myiasis, pelvic organ, cervix, hysterectomy, hygiene, maggot, uterus, prolapsed uterus

## Abstract

Myiasis of the genital organ is a rare clinical entity. It is a very disturbing condition that is linked to poor hygiene and sanitary conditions. Here, we report a case of a 62-year-old postmenopausal female who presented with the complaint of a mass coming out of the vaginal orifice for 10 years. Recently, for the last five months, the mass had become irreducible, ulcerated, and infested with maggots. A vaginal hysterectomy was performed, which provided immediate relief to the patient.

## Introduction

Infestation of living humans by maggots of flies is known as myiasis. These insects live on host tissue and body fluids [1,2]. They can cause severe infection, inflammatory reaction, and can be linked to psychiatric disturbances [3,4]. Commonly, cutaneous, ophthalmic, auricular, and nasopharyngeal myiases are seen, with genital myiasis being a rare condition [3]. Here, we present a case of myiasis of the prolapsed uterus. It is a rare case that requires attention. It is probably the first case from Pakistan to be reported in the literature.

## Case presentation

A 62-year-old postmenopausal female, para 3, presented with a complaint of a mass coming out of the vaginal orifice for the last 10 years. Initially, there were no symptoms but recently in the last five months, the mass had become irreducible, and she developed dysuria. She had no history of any previous illness or allergy. There was no family history of malignancies. There was no significant family or psychosocial history.

**Figure 1:**
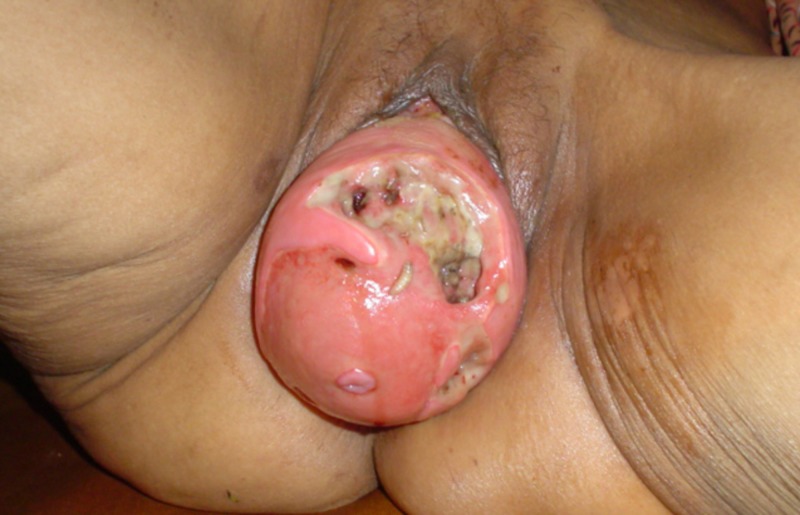
Maggot Infestation of the Prolapsed Uterus

**Figure 2:**
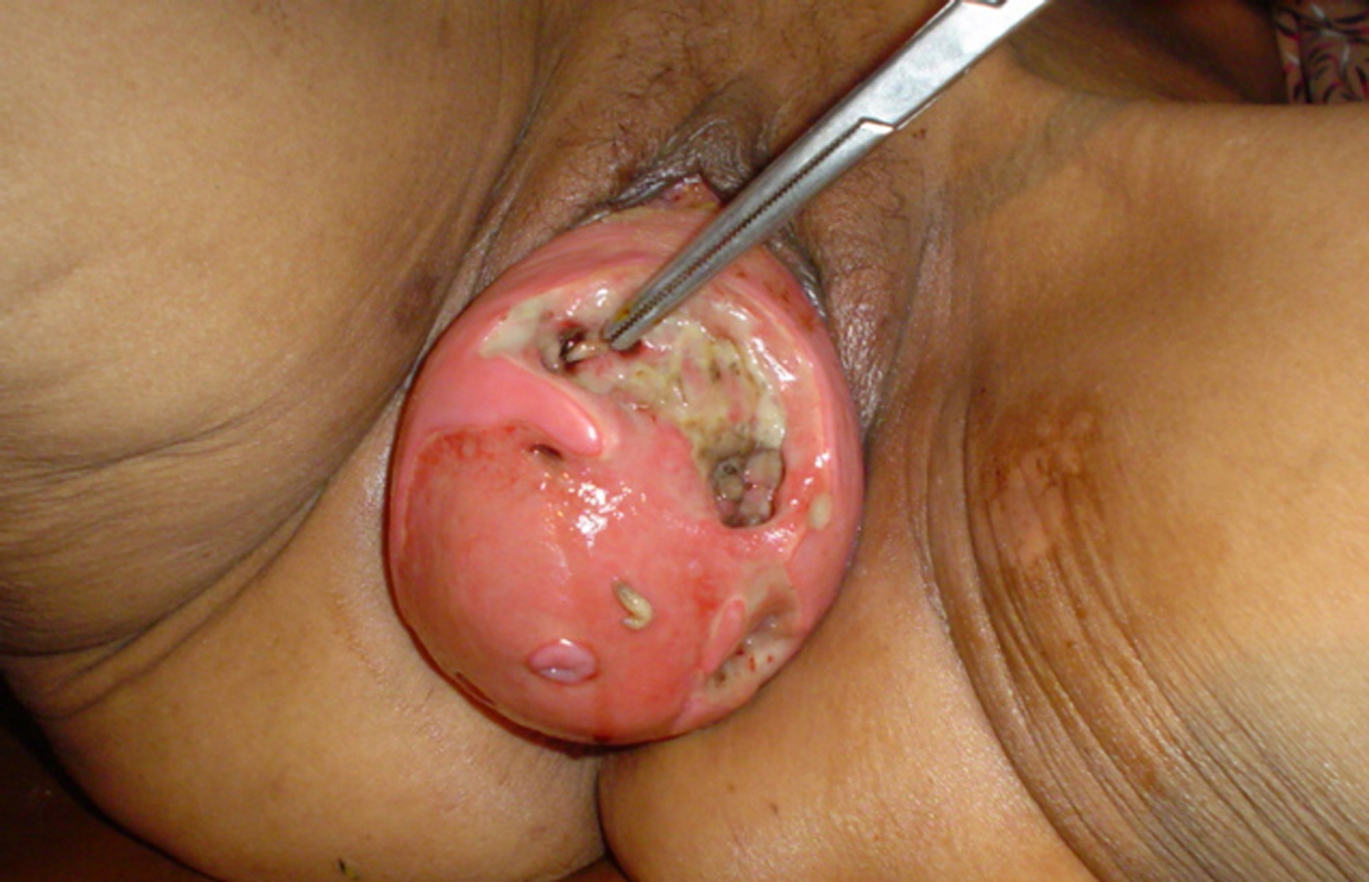
Maggot Infestation of the Prolapsed Uterus

The patient was weak and fragile. Her systemic examinations were unremarkable except for the mass coming out from the vaginal orifice (Figures 1-2).

Her vitals were normal. A genital and vaginal examination revealed an irreducible uterovaginal prolapse with maggots, larvae, and eggs. Ulcers were seen over the prolapsed mass.

Further investigation of the patient revealed that her hemoglobin, total leucocyte count (TLC), platelet count, partial thromboplastin time (PTT), activated partial thromboplastin time, blood sugar levels, and urine analysis were in normal range. Hepatitis B and C profiles were negative.

The patient was treated with analgesic and broad-spectrum antibiotics. The treatment options were presented to the patient with proper counseling. Vaginal hysterectomy was selected and informed consent was obtained. There were no intraoperative and postoperative complications. The histopathology report excluded malignancy. The symptoms resolved completely after the surgery. The patient was discharged and went back to routine life.

## Discussion

Myiasis is the parasitic infestation of a living organism by flies or insects. Commonly, they involve the nose (81%), ear (11%), tracheostomy wound (5%), gums and serous cavities (1%), face (1%). Genital myiasis is a very rare condition [1,2]. Kasinathan et al., Ray et al., and Saldarriaga et al. mention different cases of uterovaginal prolapse with myiasis, which were treated successfully [1,2,5]. Purnima et al. in their study have reported a similar condition in a middle-aged female with mental ill health [3]. Vulval myiasis has also been reported in the literature [6]. Baidya et al. have reported this entity in the female with cervical and vaginal malignancy, respectively [4]. However, in our patient, the histopathology report excluded malignancy. The ulcers were most probably because of the friction and exposure to the external environment.

Many substances like chloroform and turpentine oil (1:4), ether, hydrogen peroxide, ethylene chloride, lidocaine have been mentioned in the literature for treatment of myiasis. Removal of these larvae is difficult, challenging, and time-consuming [2]. We treated the patient with broad-spectrum antibiotics, analgesics, and performed definitive surgical treatment with vaginal hysterectomy. It provided immediate relief to the patient and she had no complication. The patient returned to normal life. We did not use anti-larval medicine as it was deemed unnecessary considering that the affected part was removed by surgery.

The major predisposing factors leading to a parasitic infestation of a living organism is old age, lack of mobility, poor hygiene, prolapsed organ, ulcerative lesion, etc [1-5]. Most of these factors were present in our patient. Good hygiene is necessary to avoid a maggot infestation. Moreover, health education in developing countries like Pakistan is very important. These measures will protect individuals from many diseases that are common in this region.

## Conclusions

Myiasis of the genital organ is a rare clinical entity. Good hygiene and proper sanitary conditions are very important for the prevention of myiasis. Vaginal hysterectomy is the definitive treatment option that can provide immediate relief to the patient.
